# Supplementary material for: The Peptide Venom Composition of the Fierce Stinging Ant Tetraponera aethiops (Formicidae: Pseudomyrmecinae)
Source: Toxins (Basel). 2019 Dec 14;11(12):732. doi: 10.3390/toxins11120732 (PMC6950161; doi:10.3390/toxins11120732)
Supplement: Supplementary file 1 [file toxins-11-00732-s001.pdf]

The Peptide Venom Composition of the Fierce Stinging Ant *Tetraponera Aethiops* (Formicidae: Pseudomyrmecinae)

Valentine Barassé, Axel Touchard, Nathan Téné, Maurice Tindo, Martin Kenne, Christophe Klopp, Alain Dejean, Elsa Bonnafé and Michel Treilhou

Table S1. Addressing table of major contigs expressed by *Tetraponera aethiops* venom glands.

	Length (kb)	Hits	Reads Per Million	%	Contig_ORF	Seq ID Name	E-value	Function
CL1015Contig1_1	448	118970	408.07	0.04				
CL1134Contig1_1	1124	122638	420.65	0.04	CL1134Contig1_1_6	sp|Q8WRF3|RL32_APIME 60S ribosomal protein L32 OS = Apis mellifera OX = 7460 GN = RpL32 PE = 2 SV = 1	2.45e-82	Translation
CL114Contig1_1	1044	189151	648.79	0.06				
CL114Contig2_1	3807	8616798	29555.53	2.96		U2-PSDTX-Ta1a Genbank Accession Number : MN607169		Venom peptide
CL1185Contig1_1	3361	775505	2659.97	0.27				
CL1193Contig1_1	7145	1682413	5770.66	0.58	CL1193Contig1_1_5	sp|Q3ZU95|PA1_VESGE Phospholipase A1 OS = Vespula germanica OX = 30212 PE = 2 SV = 1	7.81e-21	Phospholipase
					CL1193Contig1_1_6	sp|Q8K1C7|MOT14_MOUSE Monocarboxylate transporter 14 OS = Mus musculus OX = 10090 GN = Slc16a14 PE = 2 SV = 1	2.00e-37	
					CL1193Contig1_1_6	sp|Q3ZU95|PA1_VESGE Phospholipase A1 OS = Vespula germanica OX = 30212 PE = 2 SV = 1	2.42e-43	
CL120Contig1_4	2266	123353	423.1	0.04	CL120Contig1_4_3	sp|Q1HPS0|MLR_BOMMO Myosin regulatory light chain 2 OS = Bombyx mori OX = 7091 PE = 1 SV = 1	2.16e-80	Cytosqueleton
CL1275Contig1_1	3598	18623211	63877.43	6.39		U2-PSDTX-Ta1b Genbank Accession Number : MN607170		Venom peptide

CL12Contig1_1	665	239994	823.18	0.08	CL12Contig1_1_6	sp|Q962Q7|RS23_SPOFR 40S ribosomal protein S23 OS = Spodoptera frugiperda OX = 7108 GN = RpS23 PE = 2 SV = 1	6.30e-90	Translation
CL1321Contig1_1	5444	4298354	14743.31	1.47				
CL134Contig1_5	2180	103895	356.36	0.04	CL134Contig1_5_5	sp|P29341|PABP1_MOUSE Polyadenylate-binding protein 1 OS = Mus musculus OX = 10090 GN = Pabpc1 PE = 1 SV = 2	4.23e-145	RNA Maturation
CL134Contig1_6	777	162791	558.37	0.06	CL134Contig1_6_4	sp|P20965|PABPA_XENLA Polyadenylate-binding protein 1-A OS = Xenopus laevis OX = 8355 GN = pabpc1-a PE = 1 SV = 3	8.47e-97	Transcription
CL1626Contig1_2	3783	368119	1262.64	0.13	CL1626Contig1_2_1	sp|P31689|DNJA1_HUMAN DnaJ homolog subfamily A member 1 OS = Homo sapiens OX = 9606 GN = DNAJA1 PE = 1 SV = 2	7.89e-160	Protein Maturation
					CL1626Contig1_2_4	sp|Q962Q6|RS24_SPOFR 40S ribosomal protein S24 OS = Spodoptera frugiperda OX = 7108 GN = RpS24 PE = 2 SV = 1	1.20e-71	
CL1740Contig1_1	2749	15334801	52598.21	5.26				
CL1762Contig1_1	394	213658	732.84	0.07				
CL1804Contig1_1	3123	43286324	148471.66	14.85		U2-PSDTX-Ta1c Genbank Accession Numbers : MN607168		Venom peptide
CL1844Contig1_1	2272	334426	1147.08	0.11	CL1844Contig1_1_1	sp|O18640|GBLP_DROME Guanine nucleotide-binding protein subunit beta-like protein OS = Drosophila melanogaster OX = 7227 GN = Rack1 PE = 1 SV = 2	0.0	Cellular Signaling
CL1Contig165_1	4280	383730	1316.19	0.13				
CL1Contig387_1	1547	7388170	25341.35	2.53		U3-PSDTX-Ta1a Genbank Accession Numbers : MN607165		Venom peptide
CL1Contig399_4	1164	105735	362.67	0.04	CL1Contig399_4_4	sp|P08879|NDKA_DROME Nucleoside diphosphate kinase OS = Drosophila melanogaster OX = 7227 GN = awd PE = 1 SV = 3	8.15e-82	Metabolism

CL2021Contig1_1	2516	115549	396.33	0.04	CL2021Contig1_1_6	sp|Q9VBV3|TAKT_DROME Protein takeout OS = Drosophila melanogaster OX = 7227 GN = to PE = 2 SV = 1	1.26e-08	Circadian Rythm
CL2229Contig1_1	923	371493	1274.22	0.13	CL2229Contig1_1_6	sp|Q7KF90|RL31_SPOFR 60S ribosomal protein L31 OS = Spodoptera frugiperda OX = 7108 GN = RpL31 PE = 2 SV = 1	7.20e-62	Translation
CL2248Contig1_1	2074	123272	422.82	0.04				
CL235Contig1_1	5969	465480	1596.59	0.16	CL235Contig1_1_6	sp|B2D0J4|VDPP4_APIME Venom dipeptidyl peptidase 4 OS = Apis mellifera OX = 7460 PE = 1 SV = 1	0.0	Peptide Maturation
CL2441Contig1_1	764	189511	650.02	0.07	CL2441Contig1_1_3	sp|P68203|RS27A_SPOFR Ubiquitin-40S ribosomal protein S27a OS = Spodoptera frugiperda OX = 7108 PE = 2 SV = 2	1.08e-77	Protein Degradation
CL2597Contig1_2	2581	121084	415.32	0.04	CL2597Contig1_2_6	sp|Q1HDZ5|EIF3B_BOMMO Eukaryotic translation initiation factor 3 subunit B OS = Bombyx mori OX = 7091 GN = eIF3-S9 PE = 2 SV = 1	0.0	Translation
CL260Contig1_1	15246	341448	1171.16	0.12	CL260Contig1_1_2	sp|A2T929|RXRAA_DANRE Retinoic acid receptor RXR-alpha-A OS = Danio rerio OX = 7955 GN = rxraa PE = 2 SV = 2	1.05e-142	Transcription / Cytosqueleton
					CL260Contig1_1_6	sp|Q75VN3|TCTP_BOMMO Translationally-controlled tumor protein homolog OS = Bombyx mori OX = 7091 GN = Tctp PE = 2 SV = 1	3.88e-91	
CL2682Contig1_1	1709	173042	593.53	0.06	CL2682Contig1_1_1	sp|Q95ZE8|RL14_DROVI 60S ribosomal protein L14 OS = Drosophila virilis OX = 7244 GN = RpL14 PE = 3 SV = 1	3.28e-44	Translation
CL268Contig1_1	4197	301117	1032.83	0.1	CL268Contig1_1_2	sp|P62282|RS11_RAT 40S ribosomal protein S11 OS = Rattus norvegicus OX = 10116 GN = Rps11 PE = 1 SV = 3	4.09e-70	Unknown
					CL268Contig1_1_4	sp|Q7Z3D4|LYSM3_HUMAN LysM and putative peptidoglycan-binding domain-containing protein 3 OS = Homo sapiens OX = 9606 GN = LYSMD3 PE = 1 SV = 2	2.46e-12	
CL2720Contig1_1	2730	117831	404.16	0.04	CL2720Contig1_1_6	sp|O17389|TYB_CAEEL Thymosin beta OS = Caenorhabditis elegans OX = 6239 GN = tth-1 PE = 1 SV = 2	3.19e-15	Cytosqueleton

CL2890Contig1_1	2494	105857	363.09	0.04	CL2890Contig1_1_1	sp|P55828|RS20_DROME 40S ribosomal protein S20 OS = Drosophila melanogaster OX = 7227 GN = RpS20 PE = 1 SV = 1	8.78e-58	Translation
					CL2890Contig1_1_4	sp|P41374|IF2A_DROME Eukaryotic translation initiation factor 2 subunit 1 OS = Drosophila melanogaster OX = 7227 GN = eIF2alpha PE = 2 SV = 1	3.68e-146	
CL3020Contig1_1	1913	279908	960.08	0.1	CL3020Contig1_1_2	sp|Q56FG6|RL5_LYSTE 60S ribosomal protein L5 OS = Lysiphlebus testaceipes OX = 77504 GN = RpL5 PE = 2 SV = 1	2.53e-161	Translation
CL3328Contig1_1	4478	141078	483.9	0.05	CL3328Contig1_1_5	sp|Q9VFC2|SP88E_DROME Serine protease inhibitor 88Ea OS = Drosophila melanogaster OX = 7227 GN = Spn88Ea PE = 2 SV = 1	6.23e-62	Immunity
CL349Contig1_1	1286	222310	762.52	0.08				
CL3564Contig1_1	8161	1583033	5429.79	0.54	CL3564Contig1_1_3	sp|Q3ULZ2|FHDC1_MOUSE FH2 domain-containing protein 1 OS = Mus musculus OX = 10090 GN = Fhdc1 PE = 1 SV = 3	1.18e-88	Cytosqueleton
CL3611Contig1_1	2022	135922	466.21	0.05	CL3611Contig1_1_1	sp|P54985|PPIA_BLAGE Peptidyl-prolyl cis-trans isomerase OS = Blattella germanica OX = 6973 GN = CYPA PE = 2 SV = 1	3.85e-92	Protein Maturation
CL3706Contig1_1	2966	323293	1108.89	0.11	CL3706Contig1_1_2	sp|Q63159|COQ3_RAT Ubiquinone biosynthesis O-methyltransferase. mitochondrial OS = Rattus norvegicus OX = 10116 GN = Coq3 PE = 2 SV = 2	2.12e-52	Metabolism
					CL3706Contig1_1_5	sp|Q03168|ASPP_AEDAE Lysosomal aspartic protease OS = Aedes aegypti OX = 7159 GN = AAEL006169 PE = 1 SV = 2	1.09e-150	
CL3812Contig1_1	7028	214658	736.27	0.07	CL3812Contig1_1_6	sp|B1A4F7|VDDP4_VESVU Venom dipeptidyl peptidase 4 OS = Vespula vulgaris OX = 7454 PE = 1 SV = 1	7.39e-125	Peptide Maturation
CL4093Contig1_1	1360	188957	648.12	0.06	CL4093Contig1_1_2	sp|O96647|RL10_BOMMA 60S ribosomal protein L10 OS = Bombyx mandarina OX = 7092 GN = RpL10 PE = 2 SV = 1	4.54e-74	Translation

CL4128Contig1_1	2237	102496	351.56	0.04				
CL4129Contig1_1	1117	246219	844.53	0.08	CL4129Contig1_1_6	sp∣Q10416∣HYTA_APIME Hymenoptaecin OS = Apis mellifera OX = 7460 PE = 2 SV = 1	9.76e-18	Immunity
CL4173Contig1_1	3705	343497	1178.19	0.12	CL4173Contig1_1_4	sp∣P0CG71∣UBIQ1_CAEEL Polyubiquitin-A OS = Caenorhabditis elegans OX = 6239 GN = ubq-1 PE = 3 SV = 1	0.0	Protein Degradation
CL4485Contig1_1	3472	177527	608.92	0.06	CL4485Contig1_1_4	sp∣Q962R9∣RS10_SPOFR 40S ribosomal protein S10 OS = Spodoptera frugiperda OX = 7108 GN = RpS10 PE = 2 SV = 1	4.44e-70	Translation
CL4642Contig1_1	1666	709365	2433.11	0.24	CL4642Contig1_1_2	sp∣Q9W1C9∣PEB3_DROME Ejaculatory bulb-specific protein 3 OS = Drosophila melanogaster OX = 7227 GN = EbpIII PE = 2 SV = 2	1.26e-23	Secreted Protein
CL479Contig1_5	2010	2627473	9012.21	0.9	CL479Contig1_5_5	sp∣Q9U639∣HSP7D_MANSE Heat shock 70 kDa protein cognate 4 OS = Manduca sexta OX = 7130 PE = 2 SV = 1	0.0	Protein Maturation
CL479Contig1_6	1340	220142	755.08	0.08	CL479Contig1_6_5	sp∣Q9U639∣HSP7D_MANSE Heat shock 70 kDa protein cognate 4 OS = Manduca sexta OX = 7130 PE = 2 SV = 1	2.18e-106	Protein Maturation
CL4957Contig1_1	3657	172474	591.58	0.06	CL4957Contig1_1_3	sp∣A8CAG3∣RL17_PHLPP 60S ribosomal protein L17 OS = Phlebotomus papatasi OX = 29031 GN = RpL17 PE = 2 SV = 1	6.52e-100	Translation /Secretion
					CL4957Contig1_1_6	sp∣Q7L1I2∣SV2B_HUMAN Synaptic vesicle glycoprotein 2B OS = Homo sapiens OX = 9606 GN = SV2B PE = 1 SV = 1	4.67e-49	
CL5058Contig1_1	1235	279664	959.24	0.1	CL5058Contig1_1_4	sp∣Q02878∣RL6_HUMAN 60S ribosomal protein L6 OS = Homo sapiens OX = 9606 GN = RPL6 PE = 1 SV = 3	7.07e-58	Translation
CL521Contig1_1	852	886337	3040.13	0.3	CL521Contig1_1_6	sp∣P46782∣RS5_HUMAN 40S ribosomal protein S5 OS = Homo sapiens OX = 9606 GN = RPS5 PE = 1 SV = 4	7.92e-122	Translation
CL552Contig1_4	673	209357	718.09	0.07	CL552Contig1_4_6	sp∣Q962T5∣RL24_SPOFR 60S ribosomal protein L24 OS = Spodoptera frugiperda OX = 7108 GN = RpL24 PE = 2 SV = 1	2.07e-72	Translation
CL573Contig1_1	600	264962	908.82	0.09	CL573Contig1_1_2	sp∣Q6XIM8∣RS15A_DROYA 40S ribosomal protein S15a OS = Drosophila yakuba OX = 7245 GN = RpS15Aa PE = 2 SV = 3	5.18e-84	Translation

CL575Contig1_1	1770	193468	663.59	0.07				
CL5Contig7_4	1923	147949	507.46	0.05	CL5Contig7_4_2	sp|P47830|RL27A_XENLA 60S ribosomal protein L27a OS = Xenopus laevis OX = 8355 GN = rpl27a PE = 2 SV = 2	6.25e-76	RNA Maturation /Translation
					CL5Contig7_4_4	sp|Q6AXT8|SF3A2_RAT Splicing factor 3A subunit 2 OS = Rattus norvegicus OX = 10116 GN = Sf3a2 PE = 2 SV = 1	4.48e-112	
CL660Contig1_1	3987	133617	458.3	0.05	CL660Contig1_1_5	sp|Q1HRV8|ELVL1_AEDAE Elongation of very long chain fatty acids protein AAEL008004 OS = Aedes aegypti OX = 7159 GN = AAEL008004 PE = 2 SV = 2	2.28e-105	Metabolism
CL669Contig1_1	16668	180230	618.19	0.06	CL669Contig1_1_6	sp|B6RSP1|PKHA7_DANRE Pleckstrin homology domain-containing family A member 7 OS = Danio rerio OX = 7955 GN = plekha7 PE = 2 SV = 2	2.99e-37	Secretion
CL955Contig1_1	694	197268	676.63	0.07	CL955Contig1_1_6	sp|Q8WRP6|PBGP9_SOLGI Pheromone-binding protein Gp-9 OS = Solenopsis globularia littoralis OX = 176593 GN = Gp-9 PE = 3 SV = 1	3.49e-09	Chemoreception
k25_Locus_10039_Transcript_1_1	2851	152489	523.04	0.05				
k25_Locus_108_Transcript_2_5	3260	209726	719.36	0.07	k25_Locus_108_Transcript_2_5_4	sp|P29240|5NTD_DIPOM 5'-nucleotidase OS = Diplobatis ommata OX = 1870830 PE = 2 SV = 1	2.49e-71	Metabolism
					k25_Locus_108_Transcript_2_5_6	sp|Q9XZ43|5NTD_LUTLO Protein 5NUC OS = Lutzomyia longipalpis OX = 7200 GN = 5NUC PE = 1 SV = 1	1.09e-34	
k25_Locus_11_Transcript_31_1	6299	5271032	18079.59	1.81	k25_Locus_11_Transcript_31_1_4	sp|Q3ZU95|PA1_VESGE Phospholipase A1 OS = Vespula germanica OX = 30212 PE = 2 SV = 1	1.55e-19	Phospholipase
					k25_Locus_11_Transcript_31_1_6	sp|Q68KK0|PA1_SOLIN Phospholipase A1 OS = Solenopsis invicta OX = 13686 PE = 1 SV = 1	2.07e-26	
k25_Locus_1163_Transcript_8_3	710	121774	417.68	0.04	k25_Locus_1163_Transcript_8_3_5	sp|Q9NB33|RL44_OCHTR 60S ribosomal protein L44 OS = Ochlerotatus triseriatus OX = 7162 GN = RpL44 PE = 3 SV = 3	4.23e-58	Translation
k25_Locus_1168_Transcript_5_1	5138	102343	351.04	0.04	k25_Locus_1168_Transcript_5_1_1	sp|Q9CR60|GOT1B_MOUSE Vesicle transport protein GOT1B OS = Mus musculus OX = 10090 GN = Golt1b PE = 1 SV = 1	7.79e-34	Metabolism

					k25_Locus_1168_Transcript_5_1_6	sp|Q7PQV7|ADT2_ANOGA ADP.ATP carrier protein 2 OS = Anopheles gambiae OX = 7165 GN = AGAP002358 PE = 3 SV = 2	3.43e-173	
k25_Locus_1204_Transcript_6_1	2284	252383	865.67	0.09	k25_Locus_1204_Transcript_6_1_5	sp|Q4GXG7|RL18_TIMBA 60S ribosomal protein L18 OS = Timarcha balearica OX = 79517 GN = RpL18 PE = 2 SV = 1	1.45e-105	Translation
k25_Locus_1236_Transcript_1_1	1266	412434	1414.64	0.14	k25_Locus_1236_Transcript_1_1_4	sp|P41822|FRI_AEDAE Ferritin subunit OS = Aedes aegypti OX = 7159 GN = FERH PE = 1 SV = 2	1.46e-41	Metabolism
k25_Locus_126_Transcript_5_1	5493	3778623	12960.64	1.3				Metabolism
k25_Locus_1289_Transcript_17_4	1122	145672	499.65	0.05	k25_Locus_1289_Transcript_17_4_1	sp|P47830|RL27A_XENLA 60S ribosomal protein L27a OS = Xenopus laevis OX = 8355 GN = rpl27a PE = 2 SV = 2	6.25e-76	Translation
k25_Locus_1373_Transcript_14_1	2718	541864	1858.59	0.19	k25_Locus_1373_Transcript_14_1_2	sp|Q9V447|KRH2_DROME Krueppel homolog 2 OS = Drosophila melanogaster OX = 7227 GN = Kr-h2 PE = 1 SV = 1	6.94e-59	Protein Maturation /Translation
					k25_Locus_1373_Transcript_14_1_5	sp|Q5R465|RS3_PONAB 40S ribosomal protein S3 OS = Pongo abelii OX = 9601 GN = RPS3 PE = 2 SV = 1	7.22e-52	
k25_Locus_165_Transcript_2_1	833	433100	1485.53	0.15	k25_Locus_165_Transcript_2_1_1	sp|P58375|RL30_SPOFR 60S ribosomal protein L30 OS = Spodoptera frugiperda OX = 7108 GN = RpL30 PE = 3 SV = 1	3.37e-59	Translation
k25_Locus_182_Transcript_10_1	4550	136590	468.5	0.05	k25_Locus_182_Transcript_10_1_3	sp|Q7KN62|TERA_DROME Transitional endoplasmic reticulum ATPase TER94 OS = Drosophila melanogaster OX = 7227 GN = TER94 PE = 1 SV = 1	0.0	Secretion
k25_Locus_1873_Transcript_1_1	957	110742	379.84	0.04	k25_Locus_1873_Transcript_1_1_3	sp|Q8VZ67|Y4919_ARATH Uncharacterized zinc finger CCHC domain-containing protein At4g19190 OS = Arabidopsis thaliana OX = 3702 GN = At4g19190 PE = 2 SV = 1	0.54	Oxidative stress
					k25_Locus_1873_Transcript_1_1_6	sp|Q9V3P0|PRDX1_DROME Peroxiredoxin 1 OS = Drosophila melanogaster OX = 7227 GN = Jafrac1 PE = 1 SV = 1	4.60e-105	

k25_Locus_21_ Transcript_3_1	3913	58584 8	2009.45	0.2	k25_Locus_21_Tr anscript_3_1_5	sp|P29844|BIP_DROME Endoplasmic reticulum chaperone BiP OS = Drosophila melanogaster OX = 7227 GN = Hsc70-3 PE = 1 SV = 2	0.0	Protein Maturation
k25_Locus_217_ Transcript_4_2	1995	34872 4	1196.12	0.12	k25_Locus_217_T ranscript_4_2_4	sp|Q8TCT9|HM13_HUMAN Minor histocompatibility antigen H13 OS = Homo sapiens OX = 9606 GN = HM13 PE = 1 SV = 1	5.37e-131	Protein Maturation
k25_Locus_2186 _Transcript_2_3	3394	14393 4	493.69	0.05				
k25_Locus_2191 _Transcript_12_ 3	3159	13603 4	466.6	0.05	k25_Locus_2191_ Transcript_12_3_ 1	sp|Q9V778|ADAS_DROME Alkyldihydroxyacetonephosphate synthase OS = Drosophila melanogaster OX = 7227 GN = ADPS PE = 2 SV = 1	0.0	Metabolism
k25_Locus_254_ Transcript_4_4	3407	11817 1	405.33	0.04	k25_Locus_254_T ranscript_4_4_1	sp|O08623|SQSTM_RAT Sequestosome-1 OS = Rattus norvegicus OX = 10116 GN = Sqstm1 PE = 1 SV = 1	9.72e-30	Protein Degradation
k25_Locus_2739 _Transcript_4_1	2745	16403 3	562.63	0.06	k25_Locus_2739_ Transcript_4_1_5	sp|O96567|DDC_DROSI Aromatic-L-amino-acid decarboxylase OS = Drosophila simulans OX = 7240 GN = Ddc PE = 3 SV = 2	3.23e-170	Metabolism
k25_Locus_317_ Transcript_5_2	2304	14700 4	504.22	0.05	k25_Locus_317_T ranscript_5_2_5	sp|Q9VYY4|C4G15_DROME Cytochrome P450 4g15 OS = Drosophila melanogaster OX = 7227 GN = Cyp4g15 PE = 2 SV = 1	4.21e-175	Metabolism
k25_Locus_3207 _Transcript_3_1	3472	13091 4	449.03	0.04	k25_Locus_3207_ Transcript_3_1_3	sp|Q8MQS8|SP34_APIME Venom serine protease 34 OS = Apis mellifera OX = 7460 PE = 2 SV = 1	1.18e-90	Secreted Protein
k25_Locus_3286 _Transcript_1_1	894	23134 1	793.5	0.08	k25_Locus_3286_ Transcript_1_1_3	sp|Q962U0|RL13A_SPOFR 60S ribosomal protein L13a OS = Spodoptera frugiperda OX = 7108 GN = RpL13A PE = 2 SV = 1	1.91e-105	Translation
k25_Locus_3385 _Transcript_1_1	1257	48992 3	1680.43	0.17	k25_Locus_3385_ Transcript_1_1_3	sp|Q5UAP4|RSSA_BOMMO 40S ribosomal protein SA OS = Bombyx mori OX = 7091 PE = 2 SV = 1	1.39e-124	Translation
k25_Locus_3520 _Transcript_10_ 1	3033	36303 3	1245.2	0.12	k25_Locus_3520_ Transcript_10_1_ 2	sp|Q3UST5|CP089_MOUSE UPF0764 protein C16orf89 homolog OS = Mus musculus OX = 10090 PE = 2 SV = 2	1.05e-18	Unknown
k25_Locus_3576 _Transcript_3_1	1701	19106 4	655.35	0.07	k25_Locus_3576_ Transcript_3_1_5	sp|Q5R8Z6|MCFD2_PONAB Multiple coagulation factor deficiency protein 2 homolog OS = Pongo abelii OX = 9601 GN = MCFD2 PE = 2 SV = 1	2.41e-19	Secretion

k25_Locus_402_Transcript_3_1	554	167006	572.83	0.06	k25_Locus_402_Transcript_3_1_4	sp|P80455|RS12_DROME 40S ribosomal protein S12 OS = Drosophila melanogaster OX = 7227 GN = RpS12 PE = 1 SV = 2	1.23e-58	Translation
k25_Locus_4241_Transcript_1_1	674	129479	444.11	0.04	k25_Locus_4241_Transcript_1_1_4	sp|Q962Q5|RS25_SPOFR 40S ribosomal protein S25 OS = Spodoptera frugiperda OX = 7108 GN = RpS25 PE = 3 SV = 1	2.31e-43	Translation
k25_Locus_433_Transcript_9_1	4376	666502	2286.1	0.23		MKLITLFLVVVLAIFIRPLMPLQMRNTESFAEGSADAFAETNSDSIKI		Venom peptide
k25_Locus_45_Transcript_1_1	5655	1275816	4376.04	0.44	k25_Locus_45_Transcript_1_1_2	sp|P07709|NU6M_DROYA NADH-ubiquinone oxidoreductase chain 6 OS = Drosophila yakuba OX = 7245 GN = mt:ND6 PE = 3 SV = 2	6.22e-08	Metabolism
k25_Locus_4540_Transcript_2_1	2437	151912	521.06	0.05	k25_Locus_4540_Transcript_2_1_2	sp|Q10714|ACE_DROME Angiotensin-converting enzyme OS = Drosophila melanogaster OX = 7227 GN = Ance PE = 1 SV = 3	0.0	Peptide Maturation
k25_Locus_4724_Transcript_2_1	971	125734	431.27	0.04	k25_Locus_4724_Transcript_2_1_5	sp|Q962S0|RS7_SPOFR 40S ribosomal protein S7 OS = Spodoptera frugiperda OX = 7108 GN = RpS7 PE = 2 SV = 1	4.60e-100	Translation
k25_Locus_54_Transcript_3_1	2425	390874	1340.69	0.13	k25_Locus_54_Transcript_3_1_4	sp|Q9P735|FAL1_NEUCR ATP-dependent RNA helicase fal-1 OS = Neurospora crassa (strain ATCC 24698 / 74-OR23-1A / CBS 708.71 / DSM 1257 / FGSC 987) OX = 367110 GN = fal-1 PE = 3 SV = 2	1.80e-51	Translation
					k25_Locus_54_Transcript_3_1_6	sp|Q02748|IF4A_DROME Eukaryotic initiation factor 4A OS = Drosophila melanogaster OX = 7227 GN = eIF4A PE = 1 SV = 3	8.73e-84	
k25_Locus_547_Transcript_29_1	8082	144921	497.08	0.05	k25_Locus_547_Transcript_29_1_3	sp|Q767L8|MDC1_PIG Mediator of DNA damage checkpoint protein 1 OS = Sus scrofa OX = 9823 GN = MDC1 PE = 3 SV = 1	7.17e-42	DNA Repair
k25_Locus_547_Transcript_29_2	4580	136712	468.92	0.05	k25_Locus_547_Transcript_29_2_4	sp|P20735|GGT1_PIG Glutathione hydrolase 1 proenzyme OS = Sus scrofa OX = 9823 GN = GGT1 PE = 2 SV = 1	3.37e-97	Metabolism
k25_Locus_575_Transcript_4_1	3876	112409	385.56	0.04	k25_Locus_575_Transcript_4_1_2	sp|Q921M4|GOGA2_MOUSE Golgin subfamily A member 2 OS = Mus musculus OX = 10090 GN = Golga2 PE = 1 SV = 3	3.25e-47	Translation

					k25_Locus_575_Transcript_4_1_5	sp|Q69CJ9|RL35_OPHHA 60S ribosomal protein L35 OS = Ophiophagus hannah OX = 8665 GN = RPL35 PE = 2 SV = 3	4.69e-45	
k25_Locus_5867_Transcript_4_1	4342	202521	694.64	0.07	k25_Locus_5867_Transcript_4_1_5	sp|P35415|MYSP1_DROME Paramyosin. long form OS = Drosophila melanogaster OX = 7227 GN = Prm PE = 1 SV = 1	0.0	Cytosqueleton
k25_Locus_737_Transcript_1_1	1004	123141	422.37	0.04				
k25_Locus_7606_Transcript_1_1	919	441600	1514.68	0.15	k25_Locus_7606_Transcript_1_1_6	sp|Q945U1|RS15_ELAOL 40S ribosomal protein S15 OS = Elaeis oleifera OX = 80265 GN = RPS15 PE = 2 SV = 1	4.01e-77	Translation
k25_Locus_766_Transcript_35_1	5458	224052	768.5	0.08				
k25_Locus_810_Transcript_1_1	1343	417629	1432.46	0.14	k25_Locus_810_Transcript_1_1_6	sp|Q95V39|RL8_SPOFR 60S ribosomal protein L8 OS = Spodoptera frugiperda OX = 7108 GN = RpL8 PE = 2 SV = 1	3.25e-157	Translation
k25_Locus_97_Transcript_10_3	1564	1366740	4687.9	0.47	k25_Locus_97_Transcript_10_3_5	sp|Q9U639|HSP7D_MANSE Heat shock 70 kDa protein cognate 4 OS = Manduca sexta OX = 7130 PE = 2 SV = 1	0.0	Protein Maturation
k25_Locus_9770_Transcript_4_1	684	333217	1142.93	0.11				
k31_Locus_11079_Transcript_6_1	2470	105216	360.89	0.04	k31_Locus_11079_Transcript_6_1_2	sp|Q58ED9|NAA20_DANRE N-alpha-acetyltransferase 20 OS = Danio rerio OX = 7955 GN = naa20 PE = 2 SV = 1	3.15e-87	Protein Maturation
					k31_Locus_11079_Transcript_6_1_3	sp|Q5M8Y1|SPCS2_XENTR Probable signal peptidase complex subunit 2 OS = Xenopus tropicalis OX = 8364 GN = spcs2 PE = 2 SV = 1	1.72e-58	
k31_Locus_1149_Transcript_6_1	4540	255907	877.76	0.09	k31_Locus_1149_Transcript_6_1_2	sp|P9WQP7|3BHS_MYCTU 3 beta-hydroxysteroid dehydrogenase/Delta 5-->4-isomerase OS = Mycobacterium tuberculosis (strain ATCC 25618 / H37Rv) OX = 83332 GN = Rv1106c PE = 1 SV = 1	4.48e-04	Translation
					k31_Locus_1149_Transcript_6_1_6	sp|Q4GXG7|RL18_TIMBA 60S ribosomal protein L18 OS = Timarcha balearica OX = 79517 GN = RpL18 PE = 2 SV = 1	1.45e-105	

k31_Locus_1256_Transcript_1_2	1926	138848	476.25	0.05	k31_Locus_1256_Transcript_1_2_5	sp∣P70195∣PSB7_MOUSE Proteasome subunit beta type-7 OS = Mus musculus OX = 10090 GN = Psmb7 PE = 1 SV = 1	7.33e-108	Protein Degradation
k31_Locus_1299_Transcript_4_1	2874	1576787	5408.36	0.54				
k31_Locus_142_Transcript_2_1	492	340493	1167.89	0.12	k31_Locus_142_Transcript_2_1_2	sp∣Q963B7∣RL9_SPOFR 60S ribosomal protein L9 OS = Spodoptera frugiperda OX = 7108 GN = RpL9 PE = 2 SV = 1	2.22e-98	Translation
k31_Locus_1543_Transcript_1_1	2987	245729	842.85	0.08	k31_Locus_1543_Transcript_1_1_4	sp∣Q962R6∣RS13_SPOFR 40S ribosomal protein S13 OS = Spodoptera frugiperda OX = 7108 GN = RpS13 PE = 2 SV = 3	1.70e-87	Translation
k31_Locus_1653_Transcript_2_1	1702	487764	1673.03	0.17				
k31_Locus_1695_Transcript_9_1	4325	140309	481.26	0.05	k31_Locus_1695_Transcript_9_1_6	sp∣P30151∣EF1B_XENLA Elongation factor 1-beta OS = Xenopus laevis OX = 8355 GN = eef1b PE = 1 SV = 3	7.13e-75	Translation
k31_Locus_179_Transcript_1_1	5057	174936	600.03	0.06	k31_Locus_179_Transcript_1_1_4	sp∣P41824∣YBOXH_APLCA Y-box factor homolog OS = Aplysia californica OX = 6500 PE = 2 SV = 1	3.61e-42	Transcription
k31_Locus_1890_Transcript_11_1	2810	754103	2586.57	0.26	k31_Locus_1890_Transcript_11_1_2	sp∣Q5BLY4∣ICA_APIME Icarapin-like OS = Apis mellifera OX = 7460 PE = 2 SV = 1	7.08e-23	Secreted Protein
k31_Locus_1920_Transcript_1_1	866	243884	836.52	0.08	k31_Locus_1920_Transcript_1_1_4	sp∣P39018∣RS19A_DROME 40S ribosomal protein S19a OS = Drosophila melanogaster OX = 7227 GN = RpS19a PE = 1 SV = 3	2.43e-62	Translation
k31_Locus_21_Transcript_1_1	2877	385067	1320.78	0.13	k31_Locus_21_Transcript_1_1_4	sp∣P29844∣BIP_DROME Endoplasmic reticulum chaperone BiP OS = Drosophila melanogaster OX = 7227 GN = Hsc70-3 PE = 1 SV = 2	0.0	Protein Maturation
k31_Locus_2212_Transcript_4_2	1771	299249	1026.42	0.1	k31_Locus_2212_Transcript_4_2_6	sp∣P30050∣RL12_HUMAN 60S ribosomal protein L12 OS = Homo sapiens OX = 9606 GN = RPL12 PE = 1 SV = 1	2.65e-86	Translation
k31_Locus_222_Transcript_2_1	2655	106899	366.66	0.04	k31_Locus_222_Transcript_2_1_5	sp∣P13008∣RS26_DROME 40S ribosomal protein S26 OS = Drosophila melanogaster OX = 7227 GN = RpS26 PE = 1 SV = 1	3.98e-58	Translation
k31_Locus_261_Transcript_21_1	2728	4640291	15916.15	1.59	k31_Locus_261_Transcript_21_1_5	sp∣P35778∣VA3_SOLIN Venom allergen 3 OS = Solenopsis invicta OX = 13686 PE = 1 SV = 2	1.05e-74	Venom allergen

k31_Locus_263_Transcript_6_1	2222	530403	1819.28	0.18	k31_Locus_263_Transcript_6_1_5	sp|P09180|RL4_DROME 60S ribosomal protein L4 OS = Drosophila melanogaster OX = 7227 GN = RpL4 PE = 1 SV = 2	4.42e-168	Translation
k31_Locus_3_Transcript_5_1	3218	139260	477.66	0.05	k31_Locus_3_Transcript_5_1_6	sp|B4MGF8|TMEDA_DROVI Transmembrane emp24 domain-containing protein bai OS = Drosophila virilis OX = 7244 GN = bai PE = 3 SV = 1	2.19e-83	Secretion
k31_Locus_3122_Transcript_1_1	809	299719	1028.03	0.1	k31_Locus_3122_Transcript_1_1_5	sp|Q9W6Y0|RS30_ORYLA 40S ribosomal protein S30 OS = Oryzias latipes OX = 8090 GN = fau PE = 3 SV = 2	3.40e-19	Translation
k31_Locus_3178_Transcript_1_1	3098	116674	400.19	0.04	k31_Locus_3178_Transcript_1_1_4	sp|P05300|LAMP1_CHICK Lysosome-associated membrane glycoprotein 1 OS = Gallus gallus OX = 9031 GN = LAMP1 PE = 2 SV = 1	1.54e-23	Protein Degradation
k31_Locus_3301_Transcript_10_1	3031	319224	1094.94	0.11	k31_Locus_3301_Transcript_10_1_5	sp|Q3UST5|CP089_MOUSE UPF0764 protein C16orf89 homolog OS = Mus musculus OX = 10090 PE = 2 SV = 2	1.05e-18	Unknown
k31_Locus_3808_Transcript_1_1	1088	124126	425.75	0.04	k31_Locus_3808_Transcript_1_1_4	sp|P41973|SODC_DROWI Superoxide dismutase [Cu-Zn] OS = Drosophila willistoni OX = 7260 GN = Sod1 PE = 3 SV = 2	8.40e-63	Oxidative stress
k31_Locus_431_Transcript_14_2	3857	102949	353.11	0.04	k31_Locus_431_Transcript_14_2_6	sp|Q9VAF0|S39AD_DROME Zinc transporter ZIP13 homolog OS = Drosophila melanogaster OX = 7227 GN = Zip99C PE = 2 SV = 1	2.00e-52	Metabolism
k31_Locus_49_Transcript_12_1	5983	7923500	27177.53	2.72		U4-PSDTX-Ta1a Genbank Accession Numbers : MN607167		Venom peptide
k31_Locus_5020_Transcript_1_1	970	187814	644.2	0.06	k31_Locus_5020_Transcript_1_1_4	sp|P46222|RL11_DROME 60S ribosomal protein L11 OS = Drosophila melanogaster OX = 7227 GN = RpL11 PE = 1 SV = 2	3.13e-106	Translation
k31_Locus_597_Transcript_7_1	3991	1057443	3627.02	0.36	k31_Locus_597_Transcript_7_1_3	sp|P29520|EF1A_BOMMO Elongation factor 1-alpha OS = Bombyx mori OX = 7091 PE = 2 SV = 1	0.0	Translation
k31_Locus_647_Transcript_12_3	840	133193	456.85	0.05	k31_Locus_647_Transcript_12_3_3	sp|B4PEU8|RS9_DROYA 40S ribosomal protein S9 OS = Drosophila yakuba OX = 7245 GN = RpS9 PE = 2 SV = 1	7.76e-111	Translation
k31_Locus_66_Transcript_1_1	1672	1619399	5554.52	0.56	k31_Locus_66_Transcript_1_1_6	sp|Q75VN3|TCTP_BOMMO Translationally-controlled tumor protein homolog OS = Bombyx mori OX = 7091 GN = Tctp PE = 2 SV = 1	1.48e-91	Cytosqueleton

k31_Locus_725_Transcript_46_1	5204	261216	895.97	0.09				
k31_Locus_725_Transcript_46_2	3694	771833	2647.38	0.26				
k31_Locus_82_Transcript_7_2	3549	602425	2066.31	0.21	k31_Locus_82_Transcript_7_2_2	sp|Q27294|CAZ_DROME RNA-binding protein cabeza OS = Drosophila melanogaster OX = 7227 GN = caz PE = 2 SV = 2	1.71e-39	Transcription / Apoptosis
					k31_Locus_82_Transcript_7_2_6	sp|Q9D2C7|BI1_MOUSE Bax inhibitor 1 OS = Mus musculus OX = 10090 GN = Tmbim6 PE = 1 SV = 1	1.59e-55	
k31_Locus_852_Transcript_8_1	3795	201865	692.39	0.07	k31_Locus_852_Transcript_8_1_1	sp|P07602|SAP_HUMAN Prosaposin OS = Homo sapiens OX = 9606 GN = PSAP PE = 1 SV = 2	6.86e-38	Cellular Signaling
k31_Locus_853_Transcript_3_1	2089	643318	2206.57	0.22	k31_Locus_853_Transcript_3_1_3	sp|Q8TGP1|YG123_YEAST Putative uncharacterized protein YGL123C-A OS = Saccharomyces cerevisiae (strain ATCC 204508 / S288c) OX = 559292 GN = YGL123C-A PE = 5 SV = 1	8.72e-05	Translation
					k31_Locus_853_Transcript_3_1_5	sp|P31009|RS2_DROME 40S ribosomal protein S2 OS = Drosophila melanogaster OX = 7227 GN = RpS2 PE = 1 SV = 2	1.43e-131	
k31_Locus_892_Transcript_3_1	1895	337998	1159.33	0.12	k31_Locus_892_Transcript_3_1_6	sp|P19889|RLA0_DROME 60S acidic ribosomal protein P0 OS = Drosophila melanogaster OX = 7227 GN = RpLP0 PE = 1 SV = 1	2.17e-155	Translation
k31_Locus_899_Transcript_10_2	2221	112284	385.13	0.04	k31_Locus_899_Transcript_10_2_5	sp|Q58FK9|KAT3_RAT Kynurenine--oxoglutarate transaminase 3 OS = Rattus norvegicus OX = 10116 GN = Kyat3 PE = 2 SV = 1	5.44e-128	Protein Maturation
k31_Locus_984_Transcript_1_1	2900	129985	445.85	0.04	k31_Locus_984_Transcript_1_1_2	sp|Q8CBY8|DCTN4_MOUSE Dynactin subunit 4 OS = Mus musculus OX = 10090 GN = Dctn4 PE = 1 SV = 1	4.98e-101	Cytosqueleton/ Translation
					k31_Locus_984_Transcript_1_1_4	sp|C0HKA1|RS14B_DROME 40S ribosomal protein S14b OS = Drosophila melanogaster OX = 7227 GN = RpS14b PE = 2 SV = 1	7.47e-85	
k37_Locus_1_Transcript_1_2	4050	136602	468.54	0.05	k37_Locus_1_Transcript_1_2_5	sp|Q4U3L0|G3P_GLOMM Glyceraldehyde-3-phosphate dehydrogenase OS = Glossina morsitans morsitans OX = 37546 GN = Gapdh PE = 2 SV = 1	0.0	Metabolism

k37_Locus_10_Transcript_14_1	4476	541277	1856.57	0.19	k37_Locus_10_Transcript_14_1_6	sp|Q68KK0|PA1_SOLIN Phospholipase A1 OS = Solenopsis invicta OX = 13686 PE = 1 SV = 1	1.54e-51	Phospholipase
k37_Locus_1071_Transcript_2_1	2105	103746	355.85	0.04	k37_Locus_1071_Transcript_2_1_5	sp|Q4GXG7|RL18_TIMBA 60S ribosomal protein L18 OS = Timarcha balearica OX = 79517 GN = RpL18 PE = 2 SV = 1	8.75e-70	Translation
k37_Locus_1280_Transcript_9_1	9989	133891	459.24	0.05	k37_Locus_1280_Transcript_9_1_6	sp|Q24238|APH4_DROME Alkaline phosphatase 4 OS = Drosophila melanogaster OX = 7227 GN = Alp4 PE = 2 SV = 3	1.50e-66	Metabolism
k37_Locus_1441_Transcript_3_4	3432	138905	476.44	0.05	k37_Locus_1441_Transcript_3_4_6	sp|Q2PQM7|IDGF4_GLOMM Chitinase-like protein Idgf4 OS = Glossina morsitans morsitans OX = 37546 GN = Idgf4 PE = 2 SV = 1	9.23e-120	Cellular Signaling
k37_Locus_1903_Transcript_4_2	4559	154337	529.37	0.05	k37_Locus_1903_Transcript_4_2_4	sp|P28648|CD63_RAT CD63 antigen OS = Rattus norvegicus OX = 10116 GN = Cd63 PE = 1 SV = 2	3.33e-29	Cellular Signaling
k37_Locus_229_Transcript_6_1	4838	236867	812.45	0.08				
k37_Locus_2459_Transcript_1_1	1204	301429	1033.9	0.1	k37_Locus_2459_Transcript_1_1_4	sp|Q962U1|RL13_SPOFR 60S ribosomal protein L13 OS = Spodoptera frugiperda OX = 7108 GN = RpL13 PE = 2 SV = 1	6.25e-94	Translation
k37_Locus_2720_Transcript_1_1	517	158613	544.04	0.05				
k37_Locus_3057_Transcript_4_1	4291	128194	439.7	0.04				
k37_Locus_391_Transcript_6_2	4093	123879	424.9	0.04	k37_Locus_391_Transcript_6_2_6	sp|Q1HRV8|ELVL1_AEDAE Elongation of very long chain fatty acids protein AAEL008004 OS = Aedes aegypti OX = 7159 GN = AAEL008004 PE = 2 SV = 2	2.63e-105	Metabolism
k37_Locus_4169_Transcript_1_1	1410	268233	920.04	0.09	k37_Locus_4169_Transcript_1_1_3	sp|P15357|RS27A_DROME Ubiquitin-40S ribosomal protein S27a OS = Drosophila melanogaster OX = 7227 GN = RpS27A PE = 1 SV = 2	8.86e-83	Protein Degradation
k37_Locus_4367_Transcript_1_1	1554	138593	475.37	0.05	k37_Locus_4367_Transcript_1_1_5	sp|Q4GXU6|RS4_CARGR 40S ribosomal protein S4 OS = Carabus granulatus OX = 118799 GN = RpS4 PE = 2 SV = 1	3.47e-152	Translation
k37_Locus_45_Transcript_14_1	5685	26654401	91424.33	9.14		U1-PSDTX-Ta1a/U4-PSDTX-Ta1a/U5-PSDTX-Ta1a Genbank Accession Numbers : MN607166/ MN607167/ MN607171		Venom peptide

k37_Locus_472_Transcript_5_3	2292	194161	665.97	0.07	k37_Locus_472_Transcript_5_3_6	sp|Q08169|HUGA_APIME Hyaluronidase OS = Apis mellifera OX = 7460 PE = 1 SV = 1	1.46e-55	Metabolism
k37_Locus_472_Transcript_5_4	2275	268842	922.13	0.09	k37_Locus_472_Transcript_5_4_6	sp|Q08169|HUGA_APIME Hyaluronidase OS = Apis mellifera OX = 7460 PE = 1 SV = 1	5.72e-59	Metabolism
k37_Locus_49_Transcript_3_1	3765	863357	2961.31	0.3	k37_Locus_49_Transcript_3_1_5	sp|Q02748|IF4A_DROME Eukaryotic initiation factor 4A OS = Drosophila melanogaster OX = 7227 GN = eIF4A PE = 1 SV = 3	1.23e-138	Translation
k37_Locus_49_Transcript_3_2	512	327445	1123.13	0.11				
k37_Locus_540_Transcript_2_1	2408	214028	734.11	0.07	k37_Locus_540_Transcript_2_1_4	sp|Q9VBV3|TAKT_DROME Protein takeout OS = Drosophila melanogaster OX = 7227 GN = to PE = 2 SV = 1	1.26e-08	Circadian Rythm
k37_Locus_544_Transcript_9_1	2629	189263	649.17	0.06				
k37_Locus_594_Transcript_7_2	3499	168433	577.72	0.06	k37_Locus_594_Transcript_7_2_5	sp|Q9GPH3|ATFC_BOMMO Activating transcription factor of chaperone OS = Bombyx mori OX = 7091 GN = ATFC PE = 2 SV = 1	5.72e-26	Protein Maturation
k37_Locus_6671_Transcript_1_1	2238	409552	1404.76	0.14	k37_Locus_6671_Transcript_1_1_1	sp|P36241|RL19_DROME 60S ribosomal protein L19 OS = Drosophila melanogaster OX = 7227 GN = RpL19 PE = 1 SV = 2	4.25e-89	Translation
k37_Locus_795_Transcript_8_1	2389	480769	1649.03	0.16	k37_Locus_795_Transcript_8_1_2	sp|O16797|RL3_DROME 60S ribosomal protein L3 OS = Drosophila melanogaster OX = 7227 GN = RpL3 PE = 1 SV = 3	0.0	Translation
k37_Locus_87_Transcript_5_1	2675	279926	960.14	0.1	k37_Locus_87_Transcript_5_1_4	sp|P21187|PABP_DROME Polyadenylate-binding protein OS = Drosophila melanogaster OX = 7227 GN = pAbp PE = 1 SV = 3	0.0	RNA Maturation
k37_Locus_921_Transcript_7_2	1145	295798	1014.58	0.1	k37_Locus_921_Transcript_7_2_4	sp|C0HKA1|RS14B_DROME 40S ribosomal protein S14b OS = Drosophila melanogaster OX = 7227 GN = RpS14b PE = 2 SV = 1	7.47e-85	Translation
k37_Locus_9365_Transcript_5_1	3029	198228	679.92	0.07	k37_Locus_9365_Transcript_5_1_3	sp|Q5ZIR1|SHLB1_CHICK Endophilin-B1 OS = Gallus gallus OX = 9031 GN = SH3GLB1 PE = 2 SV = 1	3.22e-64	Secretion / Translation

					k37_Locus_9365_Transcript_5_1_6	sp|Q962R1|RS18_SPOFR 40S ribosomal protein S18 OS = Spodoptera frugiperda OX = 7108 GN = RpS18 PE = 2 SV = 1	9.35e-85	
k43_Locus_114_Transcript_2_1	2476	214200	734.7	0.07	k43_Locus_114_Transcript_2_1_6	sp|Q963B7|RL9_SPOFR 60S ribosomal protein L9 OS = Spodoptera frugiperda OX = 7108 GN = RpL9 PE = 2 SV = 1	3.81e-91	Translation
k43_Locus_133_Transcript_7_1	2837	800213	2744.72	0.27	k43_Locus_133_Transcript_7_1_5	sp|Q3ZU95|PA1_VESGE Phospholipase A1 OS = Vespula germanica OX = 30212 PE = 2 SV = 1	4.12e-19	Phospholipase
					k43_Locus_133_Transcript_7_1_5	sp|Q68KK0|PA1_SOLIN Phospholipase A1 OS = Solenopsis invicta OX = 13686 PE = 1 SV = 1	4.11e-15	
k43_Locus_1473_Transcript_5_1	3652	407837	1398.88	0.14	k43_Locus_1473_Transcript_5_1_3	sp|P14318|MP20_DROME Muscle-specific protein 20 OS = Drosophila melanogaster OX = 7227 GN = Mp20 PE = 2 SV = 2	9.42e-96	Cytosqueleton
k43_Locus_1496_Transcript_10_1	2917	196144	672.77	0.07				
k43_Locus_15867_Transcript_1_1	600	449378	1541.36	0.15		U2-PSDTX-Ta1a Genbank Accession Number : MN607169		Venom peptide
k43_Locus_209_Transcript_2_5	3324	118224	405.51	0.04	k43_Locus_209_Transcript_2_5_1	sp|O08623|SQSTM_RAT Sequestosome-1 OS = Rattus norvegicus OX = 10116 GN = Sqstm1 PE = 1 SV = 1	9.72e-30	Protein Degradation
k43_Locus_3217_Transcript_3_3	1201	282147	967.76	0.1	k43_Locus_3217_Transcript_3_3_4	sp|P30736|RL15_CHITE 60S ribosomal protein L15 OS = Chironomus tentans OX = 7153 GN = RpL15 PE = 3 SV = 3	1.14e-109	Translation
k43_Locus_3840_Transcript_4_2	2659	166658	571.64	0.06	k43_Locus_3840_Transcript_4_2_5	sp|Q9NV56|MRGBP_HUMAN MRG/MORF4L-binding protein OS = Homo sapiens OX = 9606 GN = MRGBP PE = 1 SV = 1	7.48e-24	Transcription
k43_Locus_530_Transcript_3_1	1144	168563	578.17	0.06	k43_Locus_530_Transcript_3_1_4	sp|Q962R9|RS10_SPOFR 40S ribosomal protein S10 OS = Spodoptera frugiperda OX = 7108 GN = RpS10 PE = 2 SV = 1	4.44e-70	Translation
k43_Locus_588_Transcript_3_1	1555	5439505	18657.45	1.87	k43_Locus_588_Transcript_3_1_3	sp|P29183|LIPP_HORSE Pancreatic triacylglycerol lipase (Fragment) OS = Equus caballus OX = 9796 GN = PNLIP PE = 1 SV = 2	8.49e-31	Phospholipase

k43_Locus_684_Transcript_1_1	1148	128005	439.06	0.04				
k43_Locus_713_Transcript_5_1	4230	116545	399.75	0.04	k43_Locus_713_Transcript_5_1_1	sp|Q14186|TFDP1_HUMAN Transcription factor Dp-1 OS = Homo sapiens OX = 9606 GN = TFDP1 PE = 1 SV = 1	1.18e-99	Transcription
k43_Locus_7363_Transcript_5_1	3050	261401	896.6	0.09	k43_Locus_7363_Transcript_5_1_2	sp|Q4GXU6|RS4_CARGR 40S ribosomal protein S4 OS = Carabus granulatus OX = 118799 GN = RpS4 PE = 2 SV = 1	2.03e-166	Translation
					k43_Locus_7363_Transcript_5_1_5	sp|A4Q9E4|TTLL2_MOUSE Probable tubulin polyglutamylase TTLL2 OS = Mus musculus OX = 10090 GN = Ttll2 PE = 2 SV = 1	9.34e-79	
k43_Locus_82_Transcript_8_5	2674	760033	2606.91	0.26	k43_Locus_82_Transcript_8_5_4	sp|Q9XZ43|5NTD_LUTLO Protein 5NUC OS = Lutzomyia longipalpis OX = 7200 GN = 5NUC PE = 1 SV = 1	9.94e-78	Metabolism
					k43_Locus_82_Transcript_8_5_5	sp|B6EWW8|V5NTD_GLOBR Snake venom 5'-nucleotidase OS = Gloydius brevicaudus OX = 259325 PE = 2 SV = 1	1.27e-39	
k49_Locus_10848_Transcript_1_1	915	150777	517.16	0.05	k49_Locus_10848_Transcript_1_1_4	sp|Q02878|RL6_HUMAN 60S ribosomal protein L6 OS = Homo sapiens OX = 9606 GN = RPL6 PE = 1 SV = 3	3.50e-44	Translation
k49_Locus_11970_Transcript_2_1	2958	108163	371	0.04	k49_Locus_11970_Transcript_2_1_2	sp|P84185|ACT5C_ANOGA Actin-5C OS = Anopheles gambiae OX = 7165 GN = Act5C PE = 2 SV = 1	0.0	Cytosqueleton
k49_Locus_1327_Transcript_9_1	7028	319087	1094.47	0.11				
k49_Locus_1493_Transcript_10_1	3416	101654	348.67	0.03	k49_Locus_1493_Transcript_10_1_2	sp|Q2T9X3|SPRY7_BOVIN SPRY domain-containing protein 7 OS = Bos taurus OX = 9913 GN = SPRYD7 PE = 2 SV = 1	1.32e-55	Protein Degradation
k49_Locus_15244_Transcript_1_1	350	104281	357.68	0.04				
k49_Locus_1637_Transcript_10_1	5305	799829	2743.41	0.27	k49_Locus_1637_Transcript_10_1_6	sp|Q24238|APH4_DROME Alkaline phosphatase 4 OS = Drosophila melanogaster OX = 7227 GN = Alp4 PE = 2 SV = 3	3.33e-121	Metabolism

k49_Locus_2359_Transcript_3_1	4026	116626	400.03	0.04	k49_Locus_2359_Transcript_3_1_3	sp|Q9DA39|LFG4_MOUSE Protein lifeguard 4 OS = Mus musculus OX = 10090 GN = Tmbim4 PE = 2 SV = 1	4.06e-56	Anti-apoptosis/ RE morphology/ Translation
					k49_Locus_2359_Transcript_3_1_5	sp|Q6PFM4|LNPB_DANRE Endoplasmic reticulum junction formation protein lunapark-B OS = Danio rerio OX = 7955 GN = lnpkb PE = 2 SV = 2	1.41e-64	
					k49_Locus_2359_Transcript_3_1_6	sp|Q95WA0|RL26_LITLI 60S ribosomal protein L26 OS = Littorina littorea OX = 31216 GN = RPL26 PE = 2 SV = 1	5.49e-68	
k49_Locus_281_Transcript_3_1	2899	1693164	5807.54	0.58	k49_Locus_281_Transcript_3_1_4	sp|Q02942|TRF_BLADI Transferrin OS = Blaberus discoidalis OX = 6981 PE = 1 SV = 1	0.0	Metabolism
k49_Locus_322_Transcript_9_3	1247	404161	1386.27	0.14				
k49_Locus_322_Transcript_9_4	2573	487718	1672.87	0.17	k49_Locus_322_Transcript_9_4_5	sp|P17892|LIPR2_MOUSE Pancreatic lipase-related protein 2 OS = Mus musculus OX = 10090 GN = Pnliprp2 PE = 1 SV = 2	1.08e-86	Phospholipase
k49_Locus_397_Transcript_1_1	2724	975919	3347.39	0.33	k49_Locus_397_Transcript_1_1_5	sp|P54399|PDI_DROME Protein disulfide-isomerase OS = Drosophila melanogaster OX = 7227 GN = Pdi PE = 2 SV = 1	0.0	Protein Maturation
k49_Locus_4240_Transcript_9_2	2005	220115	754.99	0.08	k49_Locus_4240_Transcript_9_2_2	sp|Q963B6|RL10A_SPOFR 60S ribosomal protein L10a OS = Spodoptera frugiperda OX = 7108 GN = RpL10A PE = 2 SV = 1	2.71e-119	Translation
k49_Locus_450_Transcript_3_1	2738	107051	367.18	0.04				
k49_Locus_47_Transcript_1_1	1591	282287	968.24	0.1				
k49_Locus_5739_Transcript_4_1	7855	149510	512.82	0.05	k49_Locus_5739_Transcript_4_1_1	sp|Q7M4F3|CUD2_SCHGR Endocuticle structural glycoprotein SgAbd-2 OS = Schistocerca gregaria OX = 7010 PE = 1 SV = 1	4.13e-21	Cuticule
k49_Locus_8888_Transcript_2_1	4025	115905	397.55	0.04	k49_Locus_8888_Transcript_2_1_6	sp|P14318|MP20_DROME Muscle-specific protein 20 OS = Drosophila melanogaster OX = 7227 GN = Mp20 PE = 2 SV = 2	8.21e-65	Cytosqueleton
k49_Locus_9979_Transcript_1_1	496	171592	588.56	0.06				

k55_Locus_113_Transcript_10_1	3158	1321901	4534.11	0.45	k55_Locus_113_Transcript_10_1_4	sp|Q3T126|CNIH4_BOVIN Protein cornichon homolog 4 OS = Bos taurus OX = 9913 GN = CNIH4 PE = 2 SV = 1	1.60e-39	Secretion / Phospholipase
					k55_Locus_113_Transcript_10_1_6	sp|Q68KK0|PA1_SOLIN Phospholipase A1 OS = Solenopsis invicta OX = 13686 PE = 1 SV = 1	1.43e-31	
k55_Locus_12090_Transcript_2_1	2579	103030	353.39	0.04				
k55_Locus_12339_Transcript_1_1	1043	116259	398.77	0.04	k55_Locus_12339_Transcript_1_1_5	sp|P41822|FRI_AEDAE Ferritin subunit OS = Aedes aegypti OX = 7159 GN = FERH PE = 1 SV = 2	1.34e-40	Metabolism
k55_Locus_1250_Transcript_14_3	1123	356892	1224.14	0.12	k55_Locus_1250_Transcript_14_3_1	sp|Q5G5C4|RS3A_PERAM 40S ribosomal protein S3a OS = Periplaneta americana OX = 6978 GN = Parcxpwex01 PE = 2 SV = 1	2.43e-139	Translation
k55_Locus_1331_Transcript_6_1	4877	235948	809.3	0.08				
k55_Locus_1565_Transcript_7_1	5153	687748	2358.97	0.24	k55_Locus_1565_Transcript_7_1_6	sp|Q24238|APH4_DROME Alkaline phosphatase 4 OS = Drosophila melanogaster OX = 7227 GN = Alp4 PE = 2 SV = 3	6.44e-76	Metabolism
k55_Locus_185_Transcript_11_1	1096	462590	1586.68	0.16	k55_Locus_185_Transcript_11_1_5	sp|P35778|VA3_SOLIN Venom allergen 3 OS = Solenopsis invicta OX = 13686 PE = 1 SV = 2	1.15e-71	Venom allergen
k55_Locus_2271_Transcript_5_2	1733	273202	937.08	0.09	k55_Locus_2271_Transcript_5_2_2	sp|Q9DA39|LFG4_MOUSE Protein lifeguard 4 OS = Mus musculus OX = 10090 GN = Tmbim4 PE = 2 SV = 1	4.66e-50	Translation
					k55_Locus_2271_Transcript_5_2_5	sp|Q95WA0|RL26_LITLI 60S ribosomal protein L26 OS = Littorina littorea OX = 31216 GN = RPL26 PE = 2 SV = 1	5.49e-68	
k55_Locus_2505_Transcript_11_2	3085	221164	758.59	0.08	k55_Locus_2505_Transcript_11_2_6	sp|B2D0J4|VDPP4_APIME Venom dipeptidyl peptidase 4 OS = Apis mellifera OX = 7460 PE = 1 SV = 1	2.46e-110	Peptide Maturation
k55_Locus_2784_Transcript_7_1	3736	186655	640.22	0.06				
k55_Locus_2784_Transcript_7_2	1355	386247	1324.82	0.13				
k55_Locus_2824_Transcript_2_1	1210	317588	1089.32	0.11	k55_Locus_2824_Transcript_2_1_1	sp|Q5UAP4|RSSA_BOMMO 40S ribosomal protein SA OS = Bombyx mori OX = 7091 PE = 2 SV = 1	1.39e-124	Translation

k55_Locus_38_ Transcript_120_ 1	10401	43776 71	15015.37	1.5	k55_Locus_38_Tr anscript_120_1_1	sp|Q17750|UFL1_CAEEL E3 UFM1-protein ligase 1 homolog OS = Caenorhabditis elegans OX = 6239 GN = ufl-1 PE = 3 SV = 1	0.17	RNA Maturation
					k55_Locus_38_Tr anscript_120_1_3	sp|Q0JKD0|GLT1_ORYSJ Glutamate synthase 1 [NADH]. chloroplastic OS = Oryza sativa subsp. japonica OX = 39947 GN = Os01g0681900 PE = 2 SV = 1	0.45	
					k55_Locus_38_Tr anscript_120_1_4	sp|Q09575|YRD6_CAEEL Uncharacterized protein K02A2.6 OS = Caenorhabditis elegans OX = 6239 GN = K02A2.6 PE = 4 SV = 1	2.61e-27	
					k55_Locus_38_Tr anscript_120_1_6	sp|Q09575|YRD6_CAEEL Uncharacterized protein K02A2.6 OS = Caenorhabditis elegans OX = 6239 GN = K02A2.6 PE = 4 SV = 1	2.51e-18	
					k55_Locus_38_Tr anscript_120_1_6	sp|Q5F3X4|U5S1_CHICK 116 kDa U5 small nuclear ribonucleoprotein component OS = Gallus gallus OX = 9031 GN = EFTUD2 PE = 2 SV = 1	0.0	
k55_Locus_3981 _Transcript_1_1	1058	30387 0	1042.27	0.1				
k55_Locus_44_ Transcript_1_1	1186	33698 2	1155.84	0.12	k55_Locus_44_Tr anscript_1_1_1	sp|Q75VN3|TCTP_BOMMO Translationally-controlled tumor protein homolog OS = Bombyx mori OX = 7091 GN = Tctp PE = 2 SV = 1	1.06e-85	Cytosqueleton
k55_Locus_485_ Transcript_9_1	2206	30842 25	10578.86	1.06	k55_Locus_485_T ranscript_9_1_2	sp|Q6XZB0|LIPI_HUMAN Lipase member I OS = Homo sapiens OX = 9606 GN = LIPI PE = 1 SV = 3	5.93e-25	Phospholipase
k55_Locus_485_ Transcript_9_2	1849	67902 3	2329.04	0.23				Chemoreceptio n
k55_Locus_52_ Transcript_7_1	2781	10160 3	348.5	0.03				
k55_Locus_552_ Transcript_9_1	3315	26798 3	919.18	0.09	k55_Locus_552_T ranscript_9_1_4	sp|P0CG71|UBIQ1_CAEEL Polyubiquitin-A OS = Caenorhabditis elegans OX = 6239 GN = ubq-1 PE = 3 SV = 1	0.0	Protein Degradation
k55_Locus_5854 _Transcript_3_1	7511	25160 1	862.99	0.09	k55_Locus_5854_ Transcript_3_1_3	sp|Q7M4F3|CUD2_SCHGR Endocuticle structural glycoprotein SgAbd-2 OS = Schistocerca gregaria OX = 7010 PE = 1 SV = 1	4.13e-21	Cuticule

k55_Locus_61_Transcript_6_1	2678	478123	1639.96	0.16	k55_Locus_61_Transcript_6_1_6	sp|P29341|PABP1_MOUSE Polyadenylate-binding protein 1 OS = Mus musculus OX = 10090 GN = Pabpc1 PE = 1 SV = 2	0.0	RNA Maturation
k55_Locus_6635_Transcript_3_1	785	191741	657.67	0.07				
k55_Locus_6656_Transcript_3_2	845	113578	389.57	0.04	k55_Locus_6656_Transcript_3_2_4	sp|P18101|RL40_DROME Ubiquitin-60S ribosomal protein L40 OS = Drosophila melanogaster OX = 7227 GN = RpL40 PE = 1 SV = 2	2.40e-81	Protein Degradation
k55_Locus_7083_Transcript_1_1	2980	102356	351.08	0.04	k55_Locus_7083_Transcript_1_1_5	sp|Q9VFC2|SP88E_DROME Serine protease inhibitor 88Ea OS = Drosophila melanogaster OX = 7227 GN = Spn88Ea PE = 2 SV = 1	2.55e-61	Immunity
k55_Locus_7703_Transcript_1_1	2688	1054862	3618.17	0.36	k55_Locus_7703_Transcript_1_1_1	sp|A4IHT0|FIGL1_XENTR Fidgetin-like protein 1 OS = Xenopus tropicalis OX = 8364 GN = fignl1 PE = 2 SV = 1	3.88e-134	DNA Repair
k55_Locus_8_Transcript_7_1	4349	15942157	54681.44	5.47	k55_Locus_8_Transcript_7_1_5	sp|Q3ZU95|PA1_VESGE Phospholipase A1 OS = Vespula germanica OX = 30212 PE = 2 SV = 1	2.56e-45	Phospholipase
					k55_Locus_8_Transcript_7_1_6	sp|Q3ZU95|PA1_VESGE Phospholipase A1 OS = Vespula germanica OX = 30212 PE = 2 SV = 1	4.18e-45	
k55_Locus_9803_Transcript_1_1	4289	106632	365.75	0.04	k55_Locus_9803_Transcript_1_1_6	sp|Q9UBV2|SE1L1_HUMAN Protein sel-1 homolog 1 OS = Homo sapiens OX = 9606 GN = SEL1L PE = 1 SV = 3	0.0	Protein Maturation
k55_Locus_996_Transcript_10_1	3799	416066	1427.1	0.14				
k61_Locus_10818_Transcript_1_1	1302	298160	1022.69	0.1				
k61_Locus_11296_Transcript_3_1	1613	517779	1775.98	0.18	k61_Locus_11296_Transcript_3_1_5	sp|Q94624|RS6_MANSE 40S ribosomal protein S6 OS = Manduca sexta OX = 7130 GN = RpS6 PE = 2 SV = 1	2.98e-125	Translation
k61_Locus_175_Transcript_2_1	2594	357838	1227.38	0.12	k61_Locus_175_Transcript_2_1_2	sp|P09180|RL4_DROME 60S ribosomal protein L4 OS = Drosophila melanogaster OX = 7227 GN = RpL4 PE = 1 SV = 2	3.20e-116	Translation
k61_Locus_1787_Transcript_1_1	1871	134466	461.22	0.05	k61_Locus_1787_Transcript_1_1_5	sp|Q58FK9|KAT3_RAT Kynurenine--oxoglutarate transaminase 3 OS = Rattus norvegicus OX = 10116 GN = Kyat3 PE = 2 SV = 1	5.73e-91	Protein Maturation

k61_Locus_1873_Transcript_9_2	1132	359738	1233.9	0.12	k61_Locus_1873_Transcript_9_2_6	sp|Q5G5C4|RS3A_PERAM 40S ribosomal protein S3a OS = Periplaneta americana OX = 6978 GN = Parcxpwex01 PE = 2 SV = 1	2.43e-139	Translation
k61_Locus_2007_Transcript_1_2	904	502345	1723.04	0.17	k61_Locus_2007_Transcript_1_2_6	sp|Q8WQI7|RL18A_SPOFR 60S ribosomal protein L18a OS = Spodoptera frugiperda OX = 7108 GN = RpL18A PE = 2 SV = 1	6.38e-81	Translation
k61_Locus_2081_Transcript_6_1	3183	119923	411.33	0.04	k61_Locus_2081_Transcript_6_1_1	sp|P91887|AMPN_PLUXY Aminopeptidase N OS = Plutella xylostella OX = 51655 GN = APN1 PE = 1 SV = 1	2.01e-80	Peptide Maturation
k61_Locus_2381_Transcript_1_1	3338	243580	835.48	0.08	k61_Locus_2381_Transcript_1_1_1	sp|P12919|PDGFB_FELCA Platelet-derived growth factor subunit B OS = Felis catus OX = 9685 GN = PDGFB PE = 2 SV = 1	1.57e-05	Unknown
k61_Locus_250_Transcript_6_2	2112	206620	708.7	0.07	k61_Locus_250_Transcript_6_2_6	sp|P29413|CALR_DROME Calreticulin OS = Drosophila melanogaster OX = 7227 GN = Calr PE = 1 SV = 2	0.0	Protein Maturation
k61_Locus_2935_Transcript_2_3	2338	330722	1134.37	0.11	k61_Locus_2935_Transcript_2_3_2	sp|P06603|TBA1_DROME Tubulin alpha-1 chain OS = Drosophila melanogaster OX = 7227 GN = alphaTub84B PE = 1 SV = 1	0.0	Cytosqueleton
k61_Locus_3810_Transcript_2_1	4984	304659	1044.98	0.1	k61_Locus_3810_Transcript_2_1_6	sp|Q2TBQ5|RL7A_BOVIN 60S ribosomal protein L7a OS = Bos taurus OX = 9913 GN = RPL7A PE = 2 SV = 3	7.48e-121	Translation
k61_Locus_40_Transcript_1_1	953	531612	1823.42	0.18				Cytosqueleton
k61_Locus_438_Transcript_8_1	3543	463697	1590.48	0.16	k61_Locus_438_Transcript_8_1_1	sp|P0CG71|UBIQ1_CAEEL Polyubiquitin-A OS = Caenorhabditis elegans OX = 6239 GN = ubq-1 PE = 3 SV = 1	0.0	Protein Degradation
k61_Locus_4981_Transcript_2_1	1829	183840	630.57	0.06	k61_Locus_4981_Transcript_2_1_1	sp|P14318|MP20_DROME Muscle-specific protein 20 OS = Drosophila melanogaster OX = 7227 GN = Mp20 PE = 2 SV = 2	8.73e-89	Cytosqueleton
k61_Locus_500_Transcript_6_4	2349	239087	820.07	0.08	k61_Locus_500_Transcript_6_4_6	sp|P35502|ESTF_MYZPE Esterase FE4 OS = Myzus persicae OX = 13164 PE = 1 SV = 1	2.35e-81	Unknown
k61_Locus_582_Transcript_6_1	3659	186588	639.99	0.06	k61_Locus_582_Transcript_6_1_1	sp|P12261|EF1G_ARTSA Elongation factor 1-gamma OS = Artemia salina OX = 85549 PE = 1 SV = 3	9.62e-179	Translation
k65_Locus_104_Transcript_8_1	3715	578460	1984.11	0.2	k65_Locus_104_Transcript_8_1_4	sp|Q3T126|CNIH4_BOVIN Protein cornichon homolog 4 OS = Bos taurus OX = 9913 GN = CNIH4 PE = 2 SV = 1	1.60e-39	Secretion / Phospholipase

					k65_Locus_104_Transcript_8_1_6	sp|Q3ZU95|PA1_VESGE Phospholipase A1 OS = Vespula germanica OX = 30212 PE = 2 SV = 1	1.82e-30	
k65_Locus_1147_Transcript_2_1	3286	1567170	5375.38	0.54	k65_Locus_1147_Transcript_2_1_5	sp|P13060|EF2_DROME Elongation factor 2 OS = Drosophila melanogaster OX = 7227 GN = EF2 PE = 1 SV = 4	0.0	Translation
k65_Locus_2088_Transcript_11_1	2921	166472	571	0.06	k65_Locus_2088_Transcript_11_1_2	sp|Q9V447|KRH2_DROME Krueppel homolog 2 OS = Drosophila melanogaster OX = 7227 GN = Kr-h2 PE = 1 SV = 1	6.94e-59	Protein Maturation / Translation
					k65_Locus_2088_Transcript_11_1_5	sp|Q5R465|RS3_PONAB 40S ribosomal protein S3 OS = Pongo abelii OX = 9601 GN = RPS3 PE = 2 SV = 1	7.22e-52	
k65_Locus_26_Transcript_2_1	1745	2180583	7479.38	0.75		MTSDERRSYLPLLLAVIFVLAIVHTPSVESRASADAEADAFADALAKAIANADPGILGVIARWIWKLIQILAPTAAVEVATRLGLPQK		Venom peptide
k65_Locus_4162_Transcript_4_1	1925	205899	706.23	0.07				
k65_Locus_5275_Transcript_1_1	1660	206711	709.02	0.07	k65_Locus_5275_Transcript_1_1_3	sp|P08570|RLA1_DROME 60S acidic ribosomal protein P1 OS = Drosophila melanogaster OX = 7227 GN = RpLP1 PE = 1 SV = 2	3.78e-36	Translation
k65_Locus_596_Transcript_1_1	742	195654	671.09	0.07	k65_Locus_596_Transcript_1_1_5	sp|O76756|RS8_APIME 40S ribosomal protein S8 OS = Apis mellifera OX = 7460 GN = RpS8 PE = 2 SV = 2	2.23e-136	Translation
k65_Locus_7655_Transcript_3_1	2345	333912	1145.31	0.11	k65_Locus_7655_Transcript_3_1_4	sp|P32100|RL7_DROME 60S ribosomal protein L7 OS = Drosophila melanogaster OX = 7227 GN = RpL7 PE = 1 SV = 2	1.90e-103	Translation
k65_Locus_83_Transcript_7_1	4005	113652	389.83	0.04				
k65_Locus_8694_Transcript_4_1	2406	2085397	7152.89	0.72	k65_Locus_8694_Transcript_4_1_1	sp|P35778|VA3_SOLIN Venom allergen 3 OS = Solenopsis invicta OX = 13686 PE = 1 SV = 2	1.17e-47	Venom allergen
					k65_Locus_8694_Transcript_4_1_1	sp|P35778|VA3_SOLIN Venom allergen 3 OS = Solenopsis invicta OX = 13686 PE = 1 SV = 2	9.87e-51	
k65_Locus_9888_Transcript_6_2	1228	168267	577.15	0.06	k65_Locus_9888_Transcript_6_2_6	sp|Q6EV04|RS3A_BIPLU 40S ribosomal protein S3a OS = Biphyllus lunatus OX = 197003 PE = 2 SV = 1	1.87e-142	Translation

k69_Locus_10202_Transcript_2_2	1480	134279	460.58	0.05				
k69_Locus_1049_Transcript_1_1	548	196608	674.36	0.07				
k69_Locus_110_Transcript_5_1	3341	1921234	6589.81	0.66	k69_Locus_110_Transcript_5_1_6	sp|Q3ZU95|PA1_VESGE Phospholipase A1 OS = Vespula germanica OX = 30212 PE = 2 SV = 1	8.05e-45	Phospholipase
k69_Locus_11580_Transcript_1_1	727	198452	680.69	0.07	k69_Locus_11580_Transcript_1_1_6	sp|Q75VN3|TCTP_BOMMO Translationally-controlled tumor protein homolog OS = Bombyx mori OX = 7091 GN = Tctp PE = 2 SV = 1	1.20e-45	Cytosqueleton
k69_Locus_12272_Transcript_1_1	720	244983	840.29	0.08	k69_Locus_12272_Transcript_1_1_4	sp|O96647|RL10_BOMMA 60S ribosomal protein L10 OS = Bombyx mandarina OX = 7092 GN = RpL10 PE = 2 SV = 1	3.53e-112	Translation
k69_Locus_12355_Transcript_5_1	2460	2994867	10272.36	1.03	k69_Locus_12355_Transcript_5_1_6	sp|P35778|VA3_SOLIN Venom allergen 3 OS = Solenopsis invicta OX = 13686 PE = 1 SV = 2	1.17e-47	Venom allergen
					k69_Locus_12355_Transcript_5_1_6	sp|P35778|VA3_SOLIN Venom allergen 3 OS = Solenopsis invicta OX = 13686 PE = 1 SV = 2	1.01e-71	
k69_Locus_14593_Transcript_1_1	598	286457	982.54	0.1	k69_Locus_14593_Transcript_1_1_1	sp|P05389|RLA2_DROME 60S acidic ribosomal protein P2 OS = Drosophila melanogaster OX = 7227 GN = RpLP2 PE = 1 SV = 1	2.91e-24	Translation
k69_Locus_14964_Transcript_1_1	1925	130335	447.05	0.04	k69_Locus_14964_Transcript_1_1_4	sp|P35501|ESTE_MYZPE Esterase E4 OS = Myzus persicae OX = 13164 PE = 1 SV = 1	4.99e-82	Unknown
k69_Locus_16834_Transcript_1_1	739	659139	2260.84	0.23		U2-PSDTX-Ta1c<br>Genbank Accession Numbers : MN607168		Venom peptide
k69_Locus_2100_Transcript_4_1	7317	133914	459.32	0.05	k69_Locus_2100_Transcript_4_1_1	sp|O95602|RPA1_HUMAN DNA-directed RNA polymerase I subunit RPA1 OS = Homo sapiens OX = 9606 GN = POLR1A PE = 1 SV = 2	0.0	Transcription

k69_Locus_3195_Transcript_5_1	4866	119870	411.15	0.04	k69_Locus_3195_Transcript_5_1_5	sp|Q8IZJ3|CPMD8_HUMAN C3 and PZP-like alpha-2-macroglobulin domain-containing protein 8 OS = Homo sapiens OX = 9606 GN = CPAMD8 PE = 1 SV = 2	1.14e-06	Protein Maturation
k69_Locus_4946_Transcript_1_1	2719	246026	843.87	0.08				
k69_Locus_547_Transcript_3_1	2747	23555899	80796.5	8.08				
k69_Locus_64_Transcript_10_4	3190	2483041	8516.81	0.85	k69_Locus_64_Transcript_10_4_6	sp|Q9XZ43|5NTD_LUTLO Protein 5NUC OS = Lutzomyia longipalpis OX = 7200 GN = 5NUC PE = 1 SV = 1	4.98e-123	Metabolism
k69_Locus_678_Transcript_6_1	4344	165812	568.73	0.06	k69_Locus_678_Transcript_6_1_1	sp|P07602|SAP_HUMAN Prosaposin OS = Homo sapiens OX = 9606 GN = PSAP PE = 1 SV = 2	6.11e-38	Cellular Signaling
k69_Locus_8284_Transcript_2_1	2255	704605	2416.79	0.24				
k69_Locus_87_Transcript_11_1	1120	845555	2900.25	0.29	k69_Locus_87_Transcript_11_1_2	sp|Q9BMK4|PA2_APICC Phospholipase A2 OS = Apis cerana cerana OX = 94128 PE = 2 SV = 1	9.55e-26	Phospholipase
k69_Locus_9580_Transcript_1_1	3482	620884	2129.63	0.21	k69_Locus_9580_Transcript_1_1_1	sp|B0FWD3|NU5M_AEDAE NADH-ubiquinone oxidoreductase chain 5 OS = Aedes aegypti OX = 7159 GN = mt:ND5 PE = 3 SV = 1	1.83e-60	Metabolism
					k69_Locus_9580_Transcript_1_1_3	sp|Q1HR20|NU4M_AEDAE NADH-ubiquinone oxidoreductase chain 4 OS = Aedes aegypti OX = 7159 GN = mt:ND4 PE = 2 SV = 1	3.57e-19	
